# APOE genotype moderates the relationship between LRP1 polymorphism and cognition across the Alzheimer's disease spectrum via disturbing default mode network

**DOI:** 10.1111/cns.13716

**Published:** 2021-08-12

**Authors:** Feifei Zang, Yao Zhu, Qianqian Zhang, Chang Tan, Qing Wang, Chunming Xie, Alzheimer’s Disease Neuroimaging Initiative*

**Affiliations:** ^1^ Department of Neurology Affiliated ZhongDa Hospital School of Medicine Southeast University Nanjing China; ^2^ Neuropsychiatric Institute Affiliated ZhongDa Hospital Southeast University Nanjing China

**Keywords:** Alzheimer's disease, apolipoprotein E, default mode network, low‐density lipoprotein receptor‐related protein 1

## Abstract

**Aims:**

This study aims to investigate the mechanisms by which apolipoprotein E (*APOE*) genotype modulates the relationship between low‐density lipoprotein receptor‐related protein 1 (*LRP1*) rs1799986 variant on the default mode network (DMN) and cognition in Alzheimer's disease (AD) spectrum populations.

**Methods:**

Cross‐sectional 168 subjects of AD spectrum were obtained from Alzheimer's Disease Neuroimaging Initiative database with resting‐state fMRI scans and neuropsychological scores data. Multivariable linear regression analysis was adopted to investigate the main effects and interaction of *LRP1* and disease on the DMN. Moderation and interactive analyses were performed to assess the relationships among *APOE*, *LRP1*, and cognition. A support vector machine model was used to classify AD spectrum with altered connectivity as an objective diagnostic biomarker.

**Results:**

The main effects and interaction of *LRP1* and disease were mainly focused on the core hubs of frontal‐parietal network. Several brain regions with altered connectivity were correlated with cognitive scores in *LRP1*‐T carriers, but not in non‐carriers. *APOE* regulated the effect of *LRP1* on cognitive performance. The functional connectivity of numerous brain regions within *LRP1*‐T carriers yielded strong power for classifying AD spectrum.

**Conclusion:**

These findings suggested *LRP1* could affect DMN and provided a stage‐dependent neuroimaging biomarker for classifying AD spectrum populations.

## Graphical abstract

The main effects and interaction of *LRP1* genotype and disease status focused on the core hubs of frontal‐parietal network across the Alzheimer's disease spectrum. *APOE* genotype regulated the effect of *LRP1* genotype on cognitive performance.

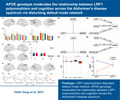

## 1 INTRODUCTION

Low‐density lipoprotein receptor‐related protein 1 (LRP1) is a large cell surface transmembrane receptor, highly expressed in the neurons, astrocytes, and vasculatures of the brain. It regulates the pathogenesis of Alzheimer's disease (AD).^1^ Reportedly, up to 50 structurally diverse proteins including β‐amyloid (Aβ) and apolipoprotein E (APOE) are ligands of LRP1.^2^ Notably, LRP1 promotes Aβ clearance, maintains synaptic integrity, and regulates lipid metabolism in the brain.^3^, ^4^ *LRP1* gene rs1799986 polymorphism in exon 3 has a silent mutation of C allele to T allele and generates three isoform genotypes including CC, TC, and TT. The T allele potentially confers a risk factor for developing sporadic AD. Nonetheless, the relationship of this variant with AD is elusive. Several previous studies reported that *LRP1* rs1799986 polymorphism was connected with late‐onset AD.^5^, ^6^, ^7^, ^8^ However, recent three genome‐wide association studies failed to discover a significant impact of this variant on AD risk.^9^, ^10^, ^11^

Despite the conflicting findings in polymorphism, one human postmortem study with brain tissues revealed that LRP1 levels from the middle frontal cortex were significantly reduced in AD patients compared with healthy controls. Also, LRP1 levels progressively decreased with the increasing age in controls, whereas a higher level of LRP1 correlated with later age of AD onset.^12^ Elsewhere, another postmortem study found significantly decreased LRP1 levels in the hippocampus of mild cognitive impairment (MCI), an early stage of AD, compared with age‐matched controls.^13^ These observations preliminarily suggest that the disrupted LRP1 levels might partly reflect brain function.

Accumulating evidence from animal studies indicates that LRP1 has been implicated in the process of Aβ and Tau pathology and related to cognitive function. Besides, LRP1 potentially acts predominantly over Aβ clearance in a mouse model of AD.^14^ In cerebral blood vessels, LRP1 importantly mediates the rapid removal of Aβ from the brain to transport across the blood‐brain barrier; also, endothelial LRP1 may be treated as a potential target for the treatment of AD.^15^ Additionally, recent research identified that knockdown *LRP1* significantly reduced tau uptake in neurons and tau propagation between neurons. This implies a master regulatory role of LRP1 in tau pathology, thereby providing a novel therapeutic target for tau‐related neurodegenerative diseases.^16^ Drug trials also have revealed that low‐dose pioglitazone ameliorates learning and memory impairment by upregulating LRP1 expression in the hippocampus.^17^ Moreover, APOE‐ε4 mediates Aβ pathology based on its neuronal receptor LRP1^18^ and *LRP1* knockout prevents the increase of Aβ pathology caused by *APOE*‐ε4 expression.^19^ Therefore, LRP1 is a common factor modulating Aβ and tau metabolism to maintain brain homeostasis.

Default mode network (DMN) is an intrinsic brain networks that most consistently exists in healthy and disease populations; its deterioration acts as a tracking tool to monitor AD progression.^20^, ^21^ Aβ accumulation preferentially commences in several core regions of DMN, including the precuneus, medial orbitofrontal, and posterior cingulate cortex, and further affected brain connectivity within DMN.^22^ Nevertheless, information on how the *LRP1* gene polymorphism affects DMN in the AD spectrum is scant, despite being the regulatory effect of LRP1 on Aβ. Additionally, considering that APOE exacerbates Aβ pathology in an LRP1‐dependent manner,^18^ investigating the role of *APOE* on the relationship between *LRP1* and cognition in AD spectrum is of importance.

Herein, we first assessed whether the *LRP1* genotype disturbed functional connectivity (FC) within DMN and affected cognitive performance across all subjects. Secondly, this work explored the relationships among *APOE*, *LRP1*, and cognition in the AD spectrum with moderation analysis. Thirdly, a support vector machine (SVM) model was employed to classify AD spectrum with the altered connectivity within DMN as an objective diagnostic biomarker, based on *LRP1* genotypes.

## 2 METHODS

### 2.1 Participants

Cross‐sectional data were downloaded from the Alzheimer's disease Neuroimaging Initiative (ADNI) database (http://adni.loni.usc.edu) before March 21, 2021. Moreover, resting‐state functional magnetic resonance imaging (rs‐fMRI) images were downloaded from ADNI‐1, ADNI‐GO, ADNI‐2, and ADNI‐3 projects. If two or more rs‐fMRI scans were performed at baseline, the first available scan was included for analysis (*n* = 184). Corrupted image sequences (*n* = 6), image quality control failure (*n* = 4), and excessive head motion (*n* = 6) were excluded. In total, 168 participants including 55 CN, 45 subjective cognitive decline (SCD), 42 MCI, and 26 mild AD participants were enrolled for the final analysis. Mini‐Mental State Examination (MMSE) was adopted as a measure of general cognition since it was available across all participants.^23^ In addition, demographic and genetic information were obtained from the ADNI database. Genetic genotyping for *APOE* and *LRP1* was performed as previously described.^24^ Participants with at least one ε4 allele were categorized into *APOE*‐ε4 carriers (*APOE* ε4^+^), while others without ε4 alleles were categorized into *APOE*‐ε4 non‐carriers (*APOE* ε4^−^). Similarly, those with at least one T allele were categorized into *LRP1*‐T carriers (*LRP1* T^+^), while others without T allele were categorized into *LRP1*‐T non‐carriers (*LRP1* T^−^). Hardy‐Weinberg equilibrium (HWE) test for each gene was calculated. Flow chart was shown in Figure S1.

### 2.2 Statistics analyses

#### 2.2.1 Demographic and neuropsychological data analysis

First, the Shapiro‐Wilk test was adopted to assess the data normality of continuous variables. A non‐parametric test was analyzed when data distributions were not normal. Levene's test was examined to assess the homogeneity of variance. One‐way analysis of variance (ANOVA) was separately used to compare the group differences of age, years of education, and MMSE scores. Non‐parametric Kruskal‐Wallis test was used if the Shapiro‐Wilk test or Levene's test *p* < 0.05. Chi‐square tests were applied to compare the group differences of gender, *APOE*‐ε4 status and *LRP1*‐T status. The significant level was set at *p *< 0.05. Post hoc analyses with Bonferroni correction (*p *< 0.05/6 = 0.0084) were essential in establishing the significance between any two groups. All statistical analyses were performed using SPSS 22.0 software (SPSS, Inc., Chicago, IL, USA).

#### 2.2.2 DMN functional connectivity analysis

A voxel‐wise one‐sample t‐test was performed on the subject‐specific maps to achieve a t‐map (DMN pattern, FDR corrected, *p *< 0.001), which was shown in Figure S2. This pattern was converted to a binary map. Of note, voxels outside of gray matter would be excluded. Subsequently, an overlap mask was generated by combining the above binary map and gray matter mask to prevent any spurious effects from white matter and ventricles. After controlling nuisance variables of age, gender, education, and *APOE* genotype, multivariable linear regression analysis was employed to investigate the effects of *LRP1* genotype, disease status, and *LRP1* × disease interaction on the DMN within the above overlap mask (3dRegAna, AFNI).

The cluster‐level threshold corrected for multiple comparisons was derived using Monte Carlo simulation of the random noise distribution in the data using the latest 3dClustSim program with the ‐acf function in AFNI [overlap DMN mask correction (39,543 voxels), voxel‐level *p* < 0.01, cluster‐level α < 0.05, κ > 113 voxels, cluster size >3051 mm^3^; https://afni.nimh.nih.gov/pub/dist/doc/program_help/3dClustSim.html]

#### 2.2.3 Behavioral correlations

After controlling covariate effects of age, gender, years of education and *APOE*‐ε4 status, linear regression analyses across all the subjects were performed. This was to investigate the correlation between behavior performance and averaged FC extracted from each region of interest identified by the main and interactive effect of *LRP1* genotype and disease status on DMN. Then, we separately analyzed the correlations of regional FC to behavior performance, specifically for *LRP1*‐T carriers and non‐carriers.

#### 2.2.4 Moderation and interactive effect analysis

A simple linear moderation effect model under PROCESS macro for SPSS (model 1)^25^ was performed to address whether *APOE* genotype moderates the genotype effect of *LRP1* on MMSE; or whether *LRP1* genotype moderates the genotype of *APOE* on MMSE, controlling for age, gender, and education. This was geared toward exploring the potential relationship between *LRP1* and *APOE*. Herein, either of the dichotomous *LRP1* genotype (*LRP1* T^+^ and T^−^) or *APOE* genotype (*APOE* ε4^+^ and ε4^−^) represents the independent variable, while another one represents moderator. MMSE represents the dependent variable. If the effect of independent variable size or sign on dependent variable varies with moderator, the effect of the independent variable on dependent variable is considered to be moderated by the moderator.

Secondly, analysis of covariance (ANCOVA), with *LRP1* genotype and *APOE* genotype, was used as dichotomous fixed factors, adjusting for potential confounding variables of age, gender and education, to illustrate the interactive effect of *LRP1* and *APOE* on MMSE across all subjects.

#### 2.2.5 Neuroimaging biomarker of FC for classifying AD spectrum disease

The extracted mean FC from each region of interest was considered a predictive variable to classify the AD spectrum among all subjects. Further, this regional FC was adopted to classify the AD spectrum in separate *LRP1*‐T carriers and non‐carriers. The receiver operating characteristic (ROC) curve was used to calculate the area under the curve (AUC) with the better predictive effect as AUC close to 1. To verify the accuracy of the classification, a leave‐one‐out cross‐validation method was used in the linear support vector machine (SVM) model implemented under MATLAB LIBSVM library^26^ and repeated 10,000 times permutation tests for the limited sample size. Unless specifically emphasized, the statistical significance was set at *p *< 0.05.

## 3 RESULTS

### 3.1 Demographic characteristics

No differences were observed in gender and education among all participants (all *p* values > 0.05). A significant decreasing trend was noted in age along the disease process, and the main difference existed between the MCI or AD groups and the CN group. While acknowledging no significance in *LRP1*‐T status, a slight marginal differential trend was noted (*p* = 0.052). Regarding *APOE*‐ε4 status, a remarkable discrepancy was noted with a tendency that the proportion of ε4 allele increased as the disease progressed. Furthermore, the MCI and AD groups showed a more significant decrease in MMSE scores; however, the SCD subjects had higher MMSE scores than the CN group. No genotype frequency deviated from HWE (LRP1, χ^2^ = 2.455, *p* = 0.117; APOE, χ^2^ = 1.580, *p* = 0.209). The characteristics of participants are summarized in Table 1.

**TABLE 1:** Demographic, genetic and clinical data across all groups

Items	CN (*n* = 55)	SCD (*n* = 45)	MCI (*n* = 42)	AD (*n* = 26)	*p* value
Age (y)	78.58 ± 6.12	75.53 ± 5.43	73.69 ± 6.20^a^	71.92 ± 7.74^b^	<0.001
Gender (M/F)	27/28	15/30	25/17	13/13	0.103^†^
Education (y)	15.96 ± 2.62	16.64 ± 2.67	15.86 ± 3.01	15.08 ± 2.64	0.082
*APOE*‐ε4 status (+/−)	14/41	12/33^d^	16/26^e^	19/7^b^	<0.001^†^
*LRP1*‐T status (+/−)	10/45	16/29	17/25	11/15	0.052^†^
MMSE	28.75 ± 1.49	29.00 ± 1.26	27.17 ± 3.81^c^	22.65 ± 3.25^bde^	<0.001

Abbreviations: AD, Alzheimer's disease; APOE, apolipoprotein E; CN, cognitively normal; LRP1, low‐density lipoprotein receptor‐related protein 1; M/F, male/female; MCI, mild cognitive impairment; MMSE, mini‐mental state examination; SCD, subjective cognitive decline.

† p values were obtained using χ^2^ test; other p values were obtained by one‐way ANOVA, but if the Shapiro‐Wilk test or the Levene's homogeneity of variance test *p* < 0.05, values were acquired by Kruskal‐Wallis test. Descriptive statistical values were presented as numbers for categorical variables and mean ± standard deviation for continuous variables. Post hoc analyses were performed with Bonferroni correction (*p* < 0.05/6 = 0.0084): ^a^, statistical difference between MCI group and CN group; ^b^, statistical difference between AD group and CN group; ^c^, statistical difference between MCI group and SCD group; ^d^, statistical difference between AD group and SCD group; ^e^, statistical difference between AD group and MCI group. No difference was found between SCD group and CN group.

### 3.2 Main and interactive effects of *LRP1* genotype and disease status on the DMN

**FIGURE 1:**
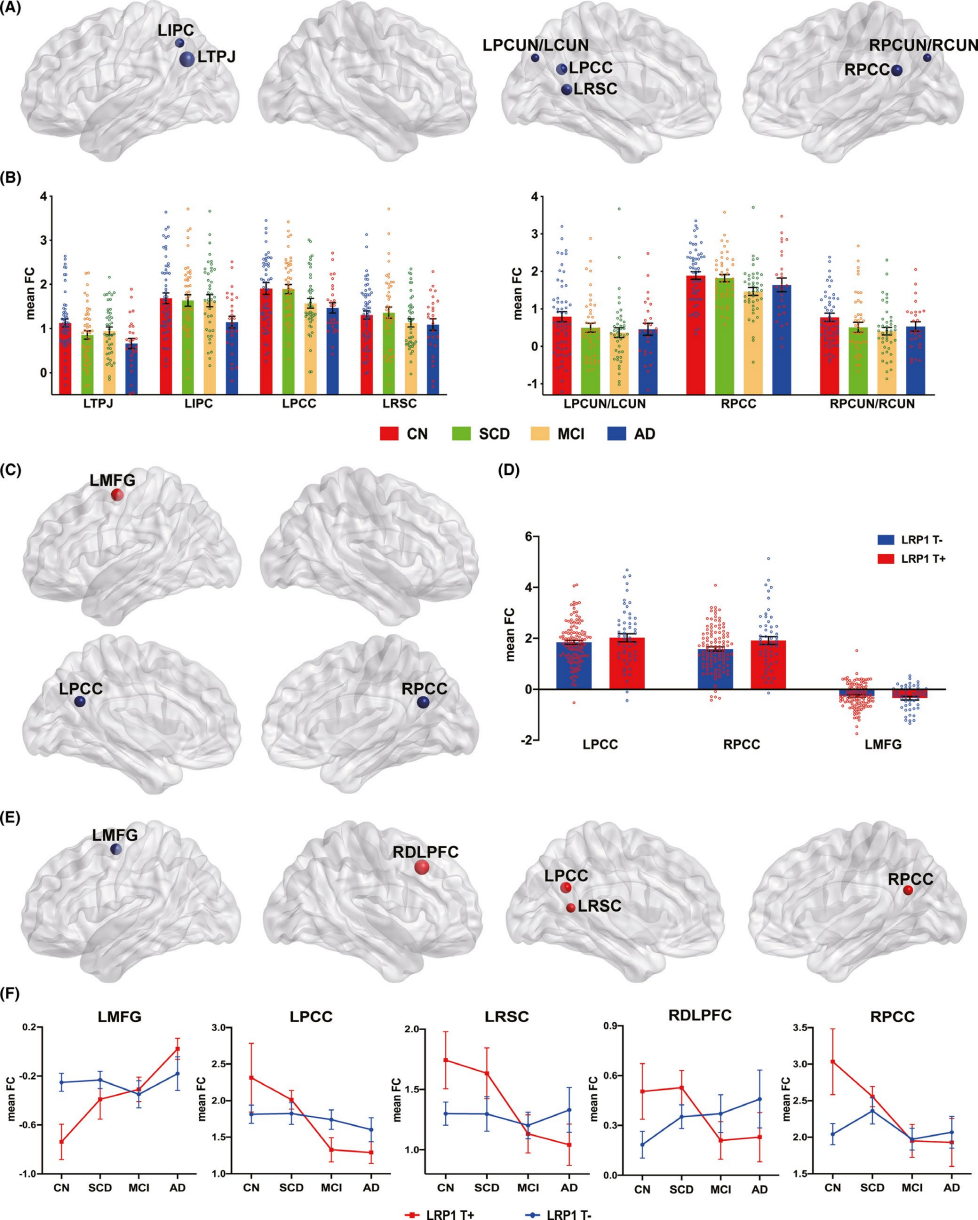
Main effect and interaction between disease status and *LRP1* genotype on the DMN across all subjects. (A) Brain regions with main effect of disease status on the DMN were identified and presented with node map. (B) Numerical representations of significant main effect of disease status on the DMN were illustrated in bar charts. (C) Brain regions significantly affected by *LRP1* genotypes on the DMN in *LRP1*‐T carriers (*LRP1* T^+^) compared with *LRP1*‐T non‐carriers (*LRP1* T^−^) were plotted with node map. (D) Numerical representations of the significant main effect of *LRP1* genotype on the DMN were described in bar charts. (E) Brain regions with significant interactive effects between *LRP1* genotype and disease on the DMN. (F) Linear trend of functional connectivity (FC) drawn via line charts represents the significant interactive effects of *LRP1* genotype and disease status on the DMN. Notably, the trajectory changes of mean FC in *LRP1*‐T carriers are opposite to *LRP1*‐T non‐carriers. Nodes’ colors and sizes in Figure A, C, and E indicate the plus and minus sign and variance of F value. Bar represents mean and standard error of mean, and each dot represents averaged functional connectivity of each participant within significant brain regions in Figure B and D. LRP1, low‐density lipoprotein receptor‐related protein 1; DMN, default mode network; CN, cognitively normal; SCD, subjective cognitive decline; MCI, mild cognitive impairment; AD, Alzheimer's disease; FC, functional connectivity; LTPJ, left temporoparietal junction; LIPC, left inferior parietal cortex; LPCC, left posterior cingulate cortex; LRSC, left retrosplenial cortex; LPCUN/LCUN, left precuneus and left cuneus; RPCC, right posterior cingulate cortex; RPCUN/RCUN, right precuneus and right cuneus; LMFG, left middle frontal gyrus; RDLPFC, right dorsal lateral prefrontal cortex.

The main effect of the disease status on the DMN was observed in the left temporoparietal joint (LTPJ), left inferior parietal cortex (LIPC), left and right posterior cingulate cortex (LPCC and RPCC), left retrosplenial cortex (LRSC), left precuneus/cuneus (LPCUN/LCUN), and right precuneus/cuneus (RPCUN/RCUN) (Figure 1A). Interestingly, the FC changes demonstrated a dynamic robust pattern along the entire disease process. The distribution pattern of FC in the LTPJ, LIPC, LPCC, and LRSC showed an inverted‐U shape, whereas FC changes in the LPCUN/LCUN, RPCC, and RPCUN/RCUN showed a U‐shape pattern (Figure 1B). The main effect of the *LRP1* genotype on the DMN was located in the left middle frontal gyrus (LMFG), LPCC, and RPCC (Figure 1C). In contrast with *LRP1*‐T non‐carriers, *LRP1*‐T carriers exhibited a stronger FC strength in these brain areas (Figure 1D).

The interactive effects of *LRP1* genotype and disease status on the DMN were also discovered in the LMFG, LPCC, RPCC, LRSC, and right dorsolateral prefrontal cortex (RDLPFC) (Figure 1E). More importantly, unlike *LRP1*‐T non‐carriers, the FC of the *LRP1*‐T carriers displayed opposite trajectory changes in these brain regions across the entire disease process, specifically between the SCD and MCI stages. The *LRP1*‐T carriers showed increased connectivity in the LMFG and decreased connectivity in the LPCC, LRSC, RDLPFC, and RPCC; nevertheless, the *LRP1*‐T non‐carriers demonstrated a relatively stable change with the progression of disease (Figure 1F). The main effects and interaction of *LRP1* genotype and disease status were majorly focused at the core hubs of the frontal‐parietal network. Comprehensive descriptions of brain regions and their FC differences among different groups are illustrated in Tables S1 and S2.

### 3.3 Relationship between altered FC and cognitive performance

**FIGURE 2:**
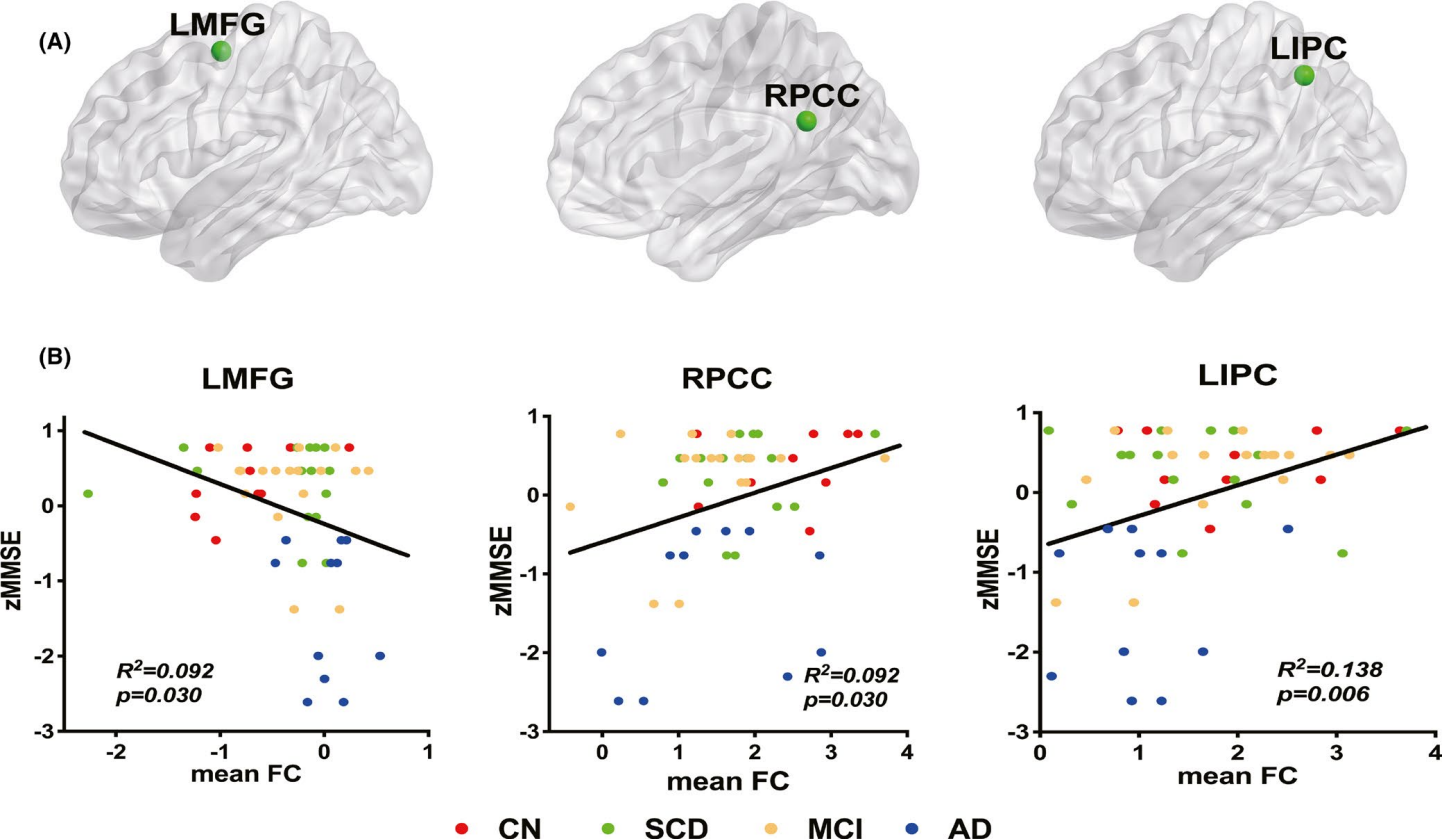
Linear regression analyses between functional connectivity and cognitive performance in the *LRP1*‐T carriers. (A) Brain nodes diagrams represented altered functional connectivity (FC) values in the regions of LMFG, RPCC, and LIPC. (B) Brain regions of LMFG, RPCC, and LIPC with altered FC could significantly affect the MMSE scores in the *LRP1*‐T carriers but not in *LRP1*‐T non‐carriers. The individual raw scores of MMSE were transformed to z scores to assure data normally distributed. Z transformation formula is z = (x ‐ μ) / σ, where x is a specific value, μ is the mean value, and σ is the standard deviation. The significance level was set at *p *< 0.05. LMFG, left middle frontal gyrus; RPCC, right posterior cingulate cortex; LIPC, left inferior parietal cortex; MMSE, mini‐mental state examination.

As shown in Figure 2, the linear regression analyses indicated that disrupted FCs in brain regions of LMFG, RPCC, and LIPC were significantly correlated with the MMSE scores across all groups in the *LRP1*‐T carriers but not in the non‐carriers. The FC of LMFG negatively correlated with the MMSE score, indicating an increased FC corresponding to a declining cognitive score. Nonetheless, the FCs of RPCC and LIPC positively correlated with MMSE scores, that is, greater FCs corresponded to higher cognitive scores.

### 3.4 Relationships among *LRP1*, *APOE*, and MMSE

**FIGURE 3:**
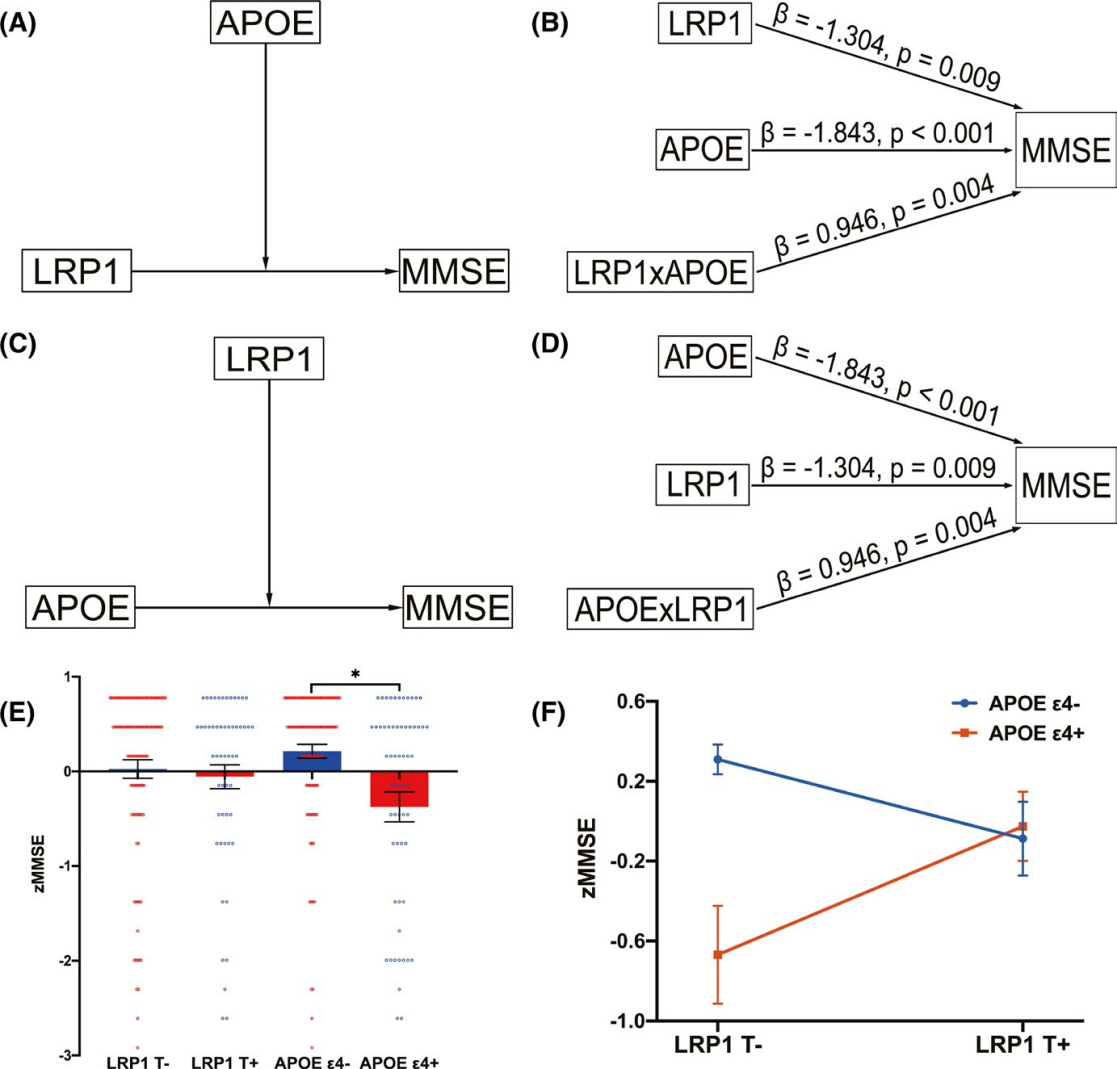
The relationships between *LRP1*, *APOE*, and MMSE across all subjects. (A) The moderation model of *APOE* genotype influencing the effect of *LRP1* genotype on MMSE scores. (B) The statistical diagram presents the effects of *LRP1* genotype, *APOE* genotype, and interaction of *LRP1* and *APOE* on MMSE scores. Moderation effect analysis revealed that *APOE* could modulate the relationship between *LRP1* and cognitive performance. (C) The moderation model of *LRP1* genotype influencing the effect of *APOE* genotype on MMSE scores. (D) The statistical diagram presents the effects of *APOE* genotype, *LRP1* genotype, and interaction of *LRP1* and *APOE* on MMSE scores. Moderation effect analysis revealed that *LRP1* could modulate the relationship between *APOE* and cognitive performance. (E) The interaction of *LRP1* genotype (T^−^/T^+^) and *APOE* genotype (ε4^−^/ε4^+^) on z‐transformed MMSE values. Bar represents mean and standard error of mean, and each dot represents z‐transformed MMSE scores of each participant within every genotype group. The main effect of *LRP1* on MMSE was insignificant (*F* = 0.510, *p* = 0.476), but that of *APOE* was significant (*F* = 6.877, *p* = 0.010). *p < 0.05. (F) The interactive effect of *LRP1* genotype (T^−^/T^+^) and *APOE* genotype (ε4^−^/ε4^+^) on MMSE scores was significant (*F* = 8.546, *p* = 0.004). Interactive effect analysis revealed that the effect of *LRP1* on MMSE was dependent on different levels of *APOE* genotype. APOE, apolipoprotein E; LRP1, low‐density lipoprotein receptor‐related protein 1; MMSE, mini‐mental state examination.

The moderation effect analysis revealed that *APOE* genotype and *LRP1* genotype regulated the effects of each other on cognitive performance across all subjects. Figure 3A shows the moderation model of *APOE* genotype affecting the effect of *LRP1* genotype on MMSE scores. As presented in Figure 3B, a relationship between *LRP1* genotype and MMSE scores was moderated by *APOE* genotype; that is because the interaction of *LRP1* genotype and *APOE* genotype significantly affected cognitive performance (β = 0.946, *p* = 0.004). Similarly, Figure 3C displays the model of the *LRP1* genotype as a moderator affecting the casual correlation of *APOE* genotype to MMSE scores. The relationship between *APOE* genotype and MMSE scores depended on the moderation of the *LRP1* genotype (Figure 3D). Specifically, *APOE* genotype and *LRP1* genotype interaction significantly affected cognition (β = 0.946, *p* = 0.004). Therefore, the effects of *LRP1* and *APOE* on cognition are moderated dependent on each other.

Furthermore, the interactive effect analysis disclosed that the effect of *LRP1* genotype on MMSE scores was dependent on different levels of *APOE* genotype. Figure 3E presents the main effect of *LRP1* on MMSE was insignificant (*F* = 0.510, *p* = 0.476), yet that of *APOE* was significant (*F* = 6.877, *p* = 0.010). As evident in Figure 3F, the interactive effect of *LRP1* genotype and *APOE* genotype on MMSE scores was important (*F* = 8.546, *p* = 0.004). Since the *LRP1* genotype shows opposing effects at both levels of *APOE* genotype, the main effect of the *LRP1* genotype is balanced out. Nonetheless, the *LRP1* genotype affects MMSE (see moderation effect above). These details of moderation and interactive effect are shown in Tables S3 and S4.

### 3.5 Stage‐dependent neuroimaging biomarker of FC for classifying AD spectrum population

**FIGURE 4:**
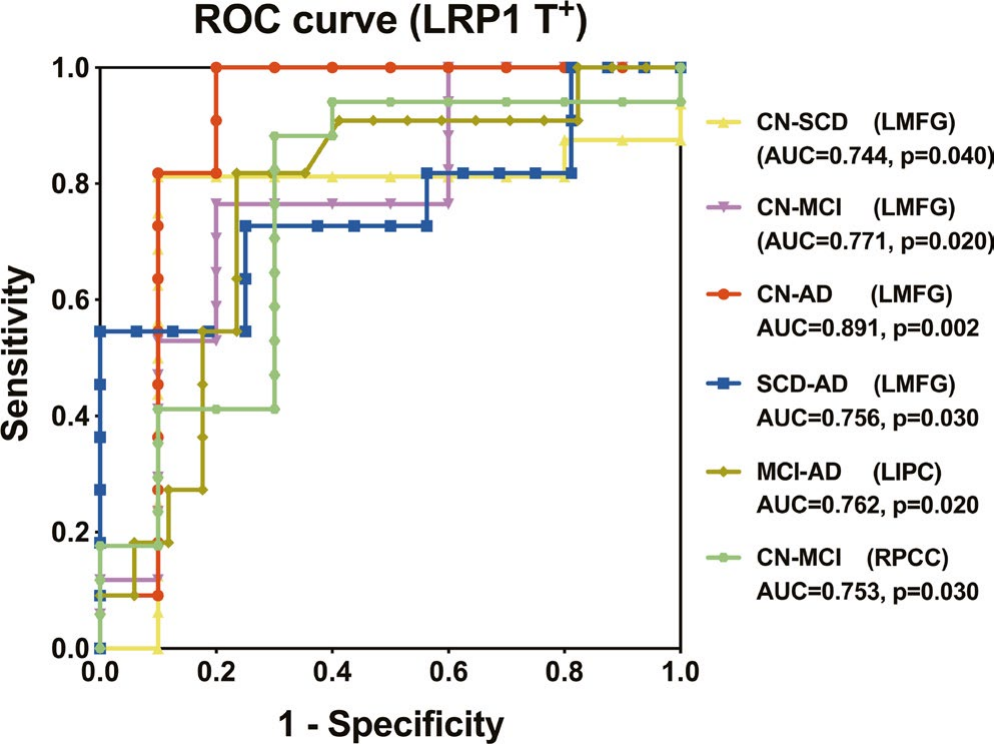
Stage‐dependent neuroimaging biomarker for classifying AD spectrum population in the *LRP1*‐T carriers. Only in the *LRP1*‐T carriers, functional connectivity (FC) values of numerous brain regions were found to be predictive variables in classifying SCD from CN (AUC = 0.744, *p* = 0.040 for LMFG), MCI from CN (AUC = 0.771, *p* = 0.020 for LMFG), AD from CN (AUC = 0.891, *p* = 0.002 for LMFG), AD from SCD (AUC = 0.756, *p* = 0.030 for LMFG), AD from MCI (AUC = 0.762, *p* = 0.020 for LIPC), MCI from CN (AUC = 0.753, *p* = 0.030 for RPCC). ROC, receiver operating characteristic; AUC, area under curve; LMFG, left middle frontal gyrus; LIPC, left inferior parietal cortex; RPCC, right posterior cingulate cortex; CN, cognitively normal; SCD, subjective cognitive decline; MCI, mild cognitive impairment; AD, Alzheimer's disease.

As displayed in Figure 4, ROC analysis indicated that FC of numerous brain regions produced a strong power for classifying different disease stages specifically in the *LRP1*‐T carriers but not in non‐carriers. In the brain area of LMFG, FC was detected as the predictive variable with AUC of 0.744 to discriminate SCD from CN, AUC of 0.771 to discriminate MCI from CN, AUC of 0.891 to discriminate AD from CN, and AUC of 0.756 to discriminate AD from SCD. Also, FC of LIPC helped discriminate AD from MCI and FC of RPCC discriminate MCI from CN. Besides, all AUC values were more than 0.7, indicating the strong power of these predictive variables to discriminate disease stages.

## 4 DISCUSSION

This study demonstrates the interactive effect of *LRP1* genotype and disease status on the DMN that primarily focused on the core hubs of the frontal‐parietal network and shows the opposite trajectory changes of FC within DMN in the *LRP1*‐T carriers compared with non‐carriers. This dichotomous pattern suggests that these vulnerable brain regions across the AD spectrum undergo different temporal and spatial patterns of progression. More importantly, we found that FCs correlated with cognitive performance in the *LRP1*‐T carriers but not in the *LRP1*‐T non‐carriers. *APOE* and *LRP1* could regulate the effect of each other on cognitive performance. Furthermore, we confirmed that the disrupted FCs potentially classify the AD spectrum population and act as a potential neuroimaging biomarker, specifically in the *LRP1*‐T carriers. These findings imply that the *LRP1* gene rs1799986 variant polymorphism consistently affects the DMN FC changes across the AD spectrum population and provides a stage‐dependent neuroimaging biomarker for early identification of the AD spectrum.

We detected the neural correlates of the *LRP1* genotype and disease status on the DMN along the AD spectrum. These brain regions belong to the frontal‐parietal network, which governs the cascade of attentional processes underlying the complex cognitive functions.^27^, ^28^, ^29^ Besides, they are vulnerable areas of AD progression, as reported formerly.^30^, ^31^, ^32^, ^33^ Interestingly, the distribution pattern of FC in the LTPJ, LIPC, LPCC, and LRSC exhibited an inverted‐U shape, suggesting that the altered FC strengths of these regions may compensate for cognitive decline in the MCI stage but decompensation occurred in the AD stage.^34^ The FC changes in the LPCUN/LCUN, RPCC, and RPCUN/RCUN manifested a U‐shape pattern, indicating a disruption in these regions‐related networks at the early stage of AD; however, compensation occurred until the AD stage.^35^, ^36^ Then, the FC strength of regions affected by the *LRP1* genotype presented an increased distributed tendency in the *LRP1*‐T carriers compared with non‐carriers. This T allele dose‐dependent distribution suggested that compensation mechanism occurred in the LPCC, RPCC, and LMFG. Notably, the posterior cingulate cortex (PCC) is an identical brain area influenced by both the *LRP1* genotype and disease status. As a crucial neural node within DMN, the PCC has been extensively studied in AD spectrum.^37^, ^38^, ^39^, ^40^ It is considered one of the earliest regions to be affected by AD based on the proposed hypothesis of cascading network failure^20^, ^21^ with early connectivity decreases^37^ and directional receiving or transmitting information.^38^

Specifically, the *LRP1*‐T carriers and non‐carriers yield opposite FC changes within DMN across the entire disease process, which might be interpreted by different neural mechanisms underlying AD progression. Notably, qualitative changes with different trajectories occurred in FC during the transition from SCD to MCI. This indicates that gene effects were mainly reflected in this selectively vulnerable transition process. Nevertheless, the genetic profile of pre‐dementia is significantly underexplored. Genetic studies in longitudinal SCD and MCI follow‐up may thus provide novel therapeutic targets and improve the existing knowledge of AD.^41^ The *LRP1*‐T carriers revealed an increased FC in the LMFG and decreased FCs in the LPCC, LRSC, RDLPFC, and RPCC, whereas, the *LRP1*‐T non‐carriers presented a relatively stable change with the progression of disease. This *LRP1* genotype‐related distribution suggested that the increased FC in LMFG likely occurs in *LRP1*‐T carriers at a higher risk of AD, which is considered compensatory reallocation of cognitive resources since it is associated with better cognitive performance.^42^ However, the decreased FCs in LPCC, LRSC, RDLPFC, and RPCC seemingly occur in *LRP1*‐T carriers at a higher risk of AD. This declining trajectory is broadly consistent with disease progression. On the other hand, the FC of *LRP1*‐T non‐carriers has a relatively stable change across four groups, indicating that the *LRP1*‐C allele might be a protective factor that prevents functional deterioration.

Moreover, the FCs of LMFG, RPCC, and LIPC correlated with MMSE scores in the *LRP1*‐T carriers but not in the non‐carriers. This indicates that the presence of risk T allele might partly influence the connection of the brain network with behavior performance. The FC of LMFG negatively correlated with MMSE, which might be explained as FC compensation for cognitive decline as above.^42^ Besides, we detected positive correlations of the RPCC and LIPC FCs with MMSE. This increased FC is broadly consistent with better cognitive performance at earlier disease stages. These findings highlight the role of the *LRP1* genotype in the intrinsic brain function of the AD spectrum population.

As mentioned above, LRP1 is a key receptor of APOE, and APOE mediates Aβ pathology depending on LRP1.^18^ This means that their corresponding genes may be inextricably linked to each other. Previous research also verified that *APOE*‐ε4 is linked to cognitive phenotypes across the AD spectrum.^43^ In the present study, two simple moderation analyses identified that the *LRP1* genotype and *APOE* genotype could regulate the effect of each other on cognitive performance. This implies the importance of removing the *APOE* effect when investigating the relationships of *LRP1* with network and cognition. Further, an interactive effect analysis was performed; this confirmed that the impact of *LRP1* on cognitive scores depends on different levels (opposing direction) of the *APOE* genotype; hence, it is balanced out (*p* > 0.05) in the interaction model. *APOE*‐ε4^+^ could promote cognitive decline even with the absence of the *LRP1*‐T allele, whereas, *APOE*‐ε4^−^ synergy with *LRP1*‐T^−^ might act a protective role for a higher cognitive function. These findings indicate that *APOE*‐ε4 is a consistent and strong genetic risk factor for AD. Besides, a strong *APOE* effect may bridge the connection between the *LRP1* genotype and the complex brain cognition as well as provide a novel outlook on the complex mechanism underlying gene‐behavior interaction.

Several studies have confirmed that brain network variables could be used as predictors in the classification of diseases.^44^, ^45^, ^46^ The FC values have been applied in distinguishing AD, MCI from CN subjects.^47^ Our group discovered that FCs of numerous brain regions including LMFG, LIPC, and RPCC could classify different disease stages of AD spectrum among all LRP1‐T carriers but not in non‐carriers. Specifically, the FC of LMFG could even classify SCD from the CN group with an AUC value of 0.744. Besides, all AUC values were more than 0.7, suggesting that FCs could yield high accuracy in classifying the AD spectrum. Also, the FCs of LMFG could discriminate SCD, MCI, AD from CN, and AD from SCD. Further, the SVM model cross‐validated this discriminating capacity. This suggests that the LMFG may be a vulnerable region susceptible to genetic influence. It is noteworthy that no intrinsic connectivity could distinguish four groups at one time. As such, the disrupted FC can serve as a stage‐dependent neuroimaging biomarker in classifying the AD spectrum.

This study has worth‐mentioning limitations that should be addressed in future research. First, the study design is cross‐sectional; thus, incorporating follow‐up data is essential to validate the predictive power of FCs for AD spectrum progression. Secondly, a recent study revealed temporal dynamic feature in AD^48^; thus, a prominent future direction might be necessary to evaluate the effect of biological factors on FC dynamics across the progression of AD. Thirdly, LRP1 mediated vascular clearance of Aβ from the brain^49^; hence, abnormal vascular function might induce cerebral hemodynamic dysregulation and further aggregate Aβ deposition. Previous studies have reported that microvascular lesions altered Aβ plaques^50^; local cerebral blood flow responses to neuronal activity were impaired in AD^51^; and neurovascular dysregulation even occurred in middle‐aged *APOE*‐ε4 carriers.^52^ Nevertheless, aerobic exercise significantly improved hippocampal blood flow in hypertensive *APOE*‐ε4 carriers, which might be beneficial for individuals at high risk of AD.^53^ Therefore, future work will focus on cerebral vascular issues and design effective intervention strategies to delay the disease progression in preclinical stage of AD. Fourthly, hypotheses such as autophagy^54^ and gut dysbiosis^55^ represented distinct signaling pathways in AD, and more genes were implicated in Aβ clearance, including *APOE*,^56^ clusterin,^57^ α2‐Macroglobulin,^58^ and triggering receptor expressed on myeloid cells 2.^59^ Thus, future research will focus on pathway‐based polygenic effects on brain networks that can better translate the underlying mechanism of AD.

## 5 CONCLUSION

In conclusion, this paper first found the *LRP1* gene rs1799986 variant polymorphism could consistently affect DMN patterns across the AD spectrum population. *APOE* regulated the effect of *LRP1* on cognitive performance. The disrupted FCs might be used as a stage‐dependent neuroimaging biomarker in classifying the AD spectrum. These findings provide novel insights into the potential mechanism underlying cognitive impairment in AD spectrum progression, and the identification of intervention targets based on genetic risk variants may offer a sneak peek at future research direction.

## CONFLICT OF INTEREST

No authors have any possible conflict of interest.

## Supporting information

Supplementary Material
