# Supplementary material for: APOE genotype moderates the relationship between LRP1 polymorphism and cognition across the Alzheimer's disease spectrum via disturbing default mode network
Source: CNS Neurosci Ther. 2021 Aug 12;27(11):1385–95. doi: 10.1111/cns.13716 (PMC8504518; doi:10.1111/cns.13716)
Supplement: Supplementary file 1 — Supplementary Material [file CNS-27-1385-s001.docx]

**Supplemental Information**

**Methods**

**Participants**

Data used in this article were obtained from the Alzheimer’s Disease Neuroimaging Initiative (ADNI) database (<http://adni.loni.usc.edu>). The ADNI was launched in 2003 as a public private partnership, led by Principal Investigator Michael W. Weiner, MD. The primary goal of the ADNI study is to track the progression of the disease using biomarkers, together with clinical measures, to assess the brain’s structure and function over the course of disease states. The ADNI was comprised of initial 5-year ADNI1 study, extended 2-year ADNIGO study, and further competitive renewals in the year of 2011 and 2016 (ADNI2 and ADNI3). This is a non-randomized natural history non-treatment study to determine the relationships among clinical, imaging, and genetic characteristics of the entire spectrum of AD. The study was approved by the Institutional Review Boards of all sites on human studies. Written informed consent was obtained from all subjects and authorized representatives before protocol-specific procedures are carried out. For up-to-date information, see [www.adni-info.org](http://www.adni-info.org). In addition, to make sure the statistical power of this study, the G*Power 3.1 software (<https://www.psychologie.hhu.de/arbeitsgruppen/allgemeine-psychologie-und-arbeitspsychologie/gpower.html>) was used^3^. When the effect size index f^2^ was set to a medium value of 0.15, and number of predictors set as 7, the total sample size will reach 103 and achieve 80% power (1-β error probability) with a significance level (α error probability) of 0.05 for the following multivariate linear regression model.

The inclusion and exclusion criteria were detailed on the ADNI website. Briefly, The CN and SCD subjects had a clinical dementia rating (CDR) score of 0, and MMSE scores between 24 and 30. While the SCD subjects represented a self-perceived cognitive decline with unimpaired performance on cognition, also known as significant memory concern (SMC) in ADNI, which may represent the first symptomatic manifestation of AD^1^. The MCI subjects should meet the criterion of MMSE scores between 24 and 30, CDR score of 0.5, objective memory loss measured through education adjusted delayed recall scores of Wechsler Memory Scale Logical Memory II (9-11 for ≥16 years; 4 for 8-15 years; ≤2 for 0-7 years), and absence of cognitive impairment in other domains. For mild AD subjects, the MMSE scores ranged from 20 to 26, a CDR equalled to 0.5 or 1.0 and met National Institute of Neurological and Communicative Diseases and Stroke/Alzheimer's Disease and Related Disorders Association (NINCDS/ADRDA) criteria for probable AD^2^. There are additional inclusion criteria that should be followed: (1) Age between 55 and 90; (2) Geriatric depression scale less than 6; (3) Hachinski score ≤4; (4) Normal visual and auditory ability adequate for neuropsychological tests. Those subjects will be excluded if they had any other significant neurodegenerative disease (Parkinson disease, Huntington’s disease), cerebrovascular disease (haemorrhage or infarction), neuropsychiatric disease (major depression, bipolar disorder within 1 year, and schizophrenia), and brain tumor, seizure, multiple sclerosis, or significant head trauma. More detailed information was available in the ADNI-2 protocol (<http://adni.loni.usc.edu/wp-content/themes/freshnews-dev-v2/documents/clinical/ADNI-2_Protocol.pdf>).

**fMRI data parameter**

All subjects were scanned under 3.0 tesla field strength of Philips MRI scanner. The rs-fMRI images were used for analysis with the following echo planar imaging sequence parameters: repetition time (TR) = 3000 ms, echo time (TE) = 30 ms, flip angle = 90°, matrix = 64×64, pixel space = 3.4 × 3.4 × 3.4 mm^3^, number of slices = 48, slice thickness = 3.4 mm, and time points = 140.

**fMRI preprocessing**

The rs-fMRI data were pre-processed using SPM12 (<https://www.fil.ion.ucl.ac.uk/spm/software/spm12/>) and RESTplusV1.24^4^ (<http://www.restfmri.net/forum/restplus>) in MATLAB 2012b (the MathWorks, Inc., Natick, Massachusetts, USA). The preprocessing steps were performed as follows: (1) the first 10 volumes were discarded for rs-fMRI signal to reach equilibrium and 130 volumes left for further analysis. (2) Due to the acquisition time differs in every slice, slice timing correction was used via temporal sinc interpolation algorithm. (3) Head motion correction was performed using a six-parameter rigid-body transformation. Subjects will be excluded with head motion excesses 2mm translation in any plane or 2°rotation in any direction. (4) The realigned images were spatially normalized to the standard Montreal Neurological Institute imaging template and were resampled to a voxel size of 3 × 3 × 3 mm^3^. (5) The normalized images were spatially smoothed using a Gaussian kernel of 6 × 6 × 6 mm.

**Extraction of intrinsic DMN pattern**

Independent component analysis (ICA) was conducted to extract spatially independent components using the GIFTv4.0b software (<http://mialab.mrn.org/software/gift/>). The subsequent processing process includes three steps: (1) Dimensionality reduction was carried out by principal component analysis to reduce complexity in computation. (2) ICA was performed to find a grouped independent component from the decomposed 30 independent components using an Infomax algorithm^5^. (3) Back reconstruction was conducted to obtain independent components for each subject and components were further converted into z scores^6^. Thus, subject-specific time courses and spatial maps were generated, and the latter would be used for further group-level inferences (see one-sample t-test below). We used the WFU toolkit (WFU PickAtlas Tool, version 2.4) to build a DMN template. Then, we applied the multiple linear regression method^7^ to sort the optimal spatial component that best matched the DMN template as the corresponding independent component pattern of DMN.

**FDR correction of DMN pattern**

False discovery rate (FDR) is referred to the proportion of discoveries that are false among all discoveries. FDR allows the researcher to tolerate a certain number of tests to be incorrectly discovered. In this study, we used a voxel-wise one-sample t-test (FDR correction, p<0.001) on subject-specific spatial maps to inference a rigorous t-map cluster that constituted the DMN pattern. We also used Random-Field Theory (RFT) correction to obtain DMN clusters and compared with that acquired by FDR correction which might raise the risk of false-positive inference. In fact, the FDR correction in the SPM package are actually GRF-FDR. The T-map of DMN pattern is first precomputed by GRF function, resulting in GRF-corrected and GRF-uncorrected maps. Then, the GRF-uncorrected map was further corrected by FDR, leading to the FDR-corrected map. At last, the DMN patterns distribution corrected by these two methods are very similar.

**DMN functional connectivity analysis**

The equation of multivariable linear regression analysis was used as follows:

$$mi=\beta0+\beta1\times disease+\beta2\times LRP1+\beta3\times\left( disease\times LRP1 \right)+\beta4\times age+\beta5\times gender+\beta6\times education+\beta7\times APOE+\varepsilon$$

The mi represents the connectivity strength of the ith voxel within DMN across entire subjects. β0 is the intercept of straight line fitted in the model; β1, β2, and β3 represent the main effects of disease, *LRP1* genotype and disease × *LRP1* interaction on the connectivity strength of the ith voxel in the DMN; β4, β5, β6, and β7 represent the main effects of covariates of no interest, including age, gender, education, and *APOE* genotype; ε is error of the above model.

**Supplemental Table S1 Descriptions of brain regions for** **LRP1 genotype × disease interaction.**

| **Brain region** | **Cluster size (mm^3^)** | **BA** | **Peak MNI Coordinates x, y, z (mm)** | **Peak F value** | **Z value** |
| --- | --- | --- | --- | --- | --- |
| **Main effect of disease** | | | | | |
| Left temporoparietal junction (LTPJ) | 6372 | 39 | -30, -63, 33 | -4.88 | 2.99 |
| Left inferior parietal cortex (LIPC) |  | 40 | -46, -57, 46 | -2.37 | 1.80 |
| Left posterior cingulate cortex (LPCC) | 4023 | 31 | -9, -57, 26 | -3.02 | 2.15 |
| Left retrosplenial cortex (LRSC) |  | 29 | -9, -53, 10 | -2.97 | 2.13 |
| Right posterior cingulate cortex (RPCC) |  | 30 | 5, -52, 25 | -3.14 | 2.21 |
| Left precuneus/left cuneus (LPCUN/LCUN) |  | 18 | -3, -78, 35 | -2.01 | 1.58 |
| Right precuneus/right cuneus (RPCUN/RCUN) |  | 18 | 5, -76, 35 | -2.00 | 1.57 |
| **Main effect of LRP1 genotype** | | | | | |
| Left posterior cingulate cortex (LPCC) | 3375 | 31 | -3, -59, 26 | -3.05 | 2.17 |
| Right posterior cingulate cortex (RPCC) |  | 30 | 3, -60, 25 | -3.53 | 2.40 |
| Left middle frontal gyrus (LMFG) | 3159 | 6 | -27, -6, 57 | 3.57 | 2.42 |
| **LRP1 genotype × disease status interaction** | | | | | |
| Right dorsal lateral prefrontal cortex (RDLPFC) | 3942 | 9 | 27, 21, 43 | 4.54 | 2.85 |
| Left middle frontal gyrus (LMFG) | 3105 | 6 | -27, -6, 57 | -3.13 | 2.21 |
| Left posterior cingulate cortex (LPCC) | 3051 | 31 | -9, -57, 27 | 3.04 | 2.16 |
| Left retrosplenial cortex (LRSC) |  | 29 | -9, -53, 11 | 2.35 | 1.78 |
| Right posterior cingulate cortex (RPCC) |  | 30 | 3, -58, 25 | 2.56 | 1.90 |

**Note**: Cluster size is in mm^3^. **Abbreviations**: BA, Brodmann’s area; MNI, Montreal Neurological Institute; LTPJ, Left temporoparietal junction; LIPC, Left inferior parietal cortex; LPCC, Left posterior cingulate cortex; LRSC, Left retrosplenial cortex; LPCUN/LCUN, Left precuneus and left cuneus; RPCC, Right posterior cingulate cortex; RPCUN/RCUN, Right precuneus and right cuneus; LMFG, Left middle frontal gyrus**;** RDLPFC, Right dorsal lateral prefrontal cortex.

**Supplemental Table S2 Group differences of brain regions for LRP1 genotype × disease interaction.**

| **Brain regions** | **Main effect of disease** | | | | | | | |
| --- | --- | --- | --- | --- | --- | --- | --- | --- |
|  | **CN** | | **SCD** | | **MCI** | | **AD** | |
| LTPJ | 1.13±0.66 | | 0.85±0.63 | | 0.95±0.58 | | 0.66±0.59 | |
| LIPC | 1.69±0.87 | | 1.64±0.85 | | 1.63±0.87 | | 1.15±0.69 | |
| LPCC | 1.90±0.99 | | 1.89±0.72 | | 1.57±0.70 | | 1.47±0.59 | |
| LRSC | 1.31±0.69 | | 1.36±0.84 | | 1.13±0.60 | | 1.09±0.67 | |
| RPCC | 1.89±0.76 | | 1.82±0.64 | | 1.46±0.69 | | 1.64±0.93 | |
| LPCUN/LCUN | 0.80±1.01 | | 0.50±0.80 | | 0.37±0.84 | | 0.46±0.82 | |
| RPCUN/RCUN | 0.78±0.81 | | 0.51±0.90 | | 0.41±0.67 | | 0.53±0.64 | |
|  | **Main effect of LRP1 genotype** | | | | | | | |
|  | **LRP1 T-** | | | | **LRP1 T+** | | | |
| LPCC | 1.84±0.82 | | | | 2.02±1.17 | | | |
| RPCC | 1.58±0.86 | | | | 1.92±1.12 | | | |
| LMFG | -0.26±0.48 | | | | -0.29±0.50 | | | |
|  | **LRP1 genotype × disease status interaction** | | | | | | | |
|  | **CN** | | **SCD** | | **MCI** | | **AD** | |
|  | **LRP1 T-** | **LRP1 T+** | **LRP1 T-** | **LRP1 T+** | **LRP1 T-** | **LRP1 T+** | **LRP1 T-** | **LRP1 T+** |
| RDLPFC | 0.18±0.54 | 0.51±0.53 | 0.35±0.39 | 0.53±0.41 | 0.37±0.57 | 0.21±0.47 | 0.46±0.68 | 0.23±0.49 |
| LMFG | -0.25±0.49 | -0.74±0.46 | -0.23±0.38 | -0.39±0.65 | -0.35±0.55 | -0.31±0.41 | -0.18±0.53 | 0.02±0.28 |
| LPCC | 1.81±0.84 | 2.31±1.48 | 1.82±0.81 | 2.01±0.52 | 1.74±0.67 | 1.33±0.68 | 1.60±0.63 | 1.29±0.50 |
| LRSC | 1.30±0.65 | 1.74±0.75 | 1.30±0.77 | 1.63±0.84 | 1.20±0.55 | 1.13±0.65 | 1.33±0.72 | 1.04±0.57 |
| RPCC | 2.04±0.98 | 3.03±1.43 | 2.36±0.97 | 2.56±0.55 | 1.97±0.75 | 1.95±0.93 | 2.07±0.85 | 1.93±1.09 |

**Note:** Numerous values represented the mean ± standard deviation of functional connectivity for significant brain regions of main and interactive effect of LRP1 genotype and disease.

**Supplemental Table S3 Detailed information of moderation effect analysis.**

| **Variables** | ***β*** | ***p*** | ***95% CI*** | ***F*** | ***R^2^*** |
| --- | --- | --- | --- | --- | --- |
|  | **X = LRP1, M = APOE, Y = MMSE,** **C = Age, Gender, Education** | | | | |
| LRP1 | -1.304 | 0.009 | -2.276, -0.332 |  |  |
| APOE | -1.843 | <0.001 | -2.779, -0.908 |  |  |
| LRP1*APOE | 0.946 | 0.004 | 0.307, 1.585 | 8.546 | 0.045 |
| Age | 0.011 | 0.350 | -0.012, 0.033 |  |  |
| Gender | 0.177 | 0.246 | -0.124, 0.478 |  |  |
| Education | 0.050 | 0.073 | -0.005, 0.104 |  |  |
|  | **X = APOE, M = LRP1, Y = MMSE, C = Age, Gender, Education** | | | | |
| APOE | -1.843 | <0.001 | -2.779, -0.908 |  |  |
| LRP1 | -1.304 | 0.009 | -2.276, -0.332 |  |  |
| APOE*LRP1 | 0.946 | 0.004 | 0.307, 1.585 | 8.546 | 0.045 |
| Age | 0.011 | 0.350 | -0.012, 0.033 |  |  |
| Gender | 0.177 | 0.246 | -0.124, 0.478 |  |  |
| Education | 0.050 | 0.073 | -0.005, 0.104 |  |  |

**Note:** The X refers to independent variable, M represents the moderator variable, Y shows the dependent variable, and C indicates the covariate variables of no interest, with each model examined separately. The values for each moderation effect model represent the regression coefficient (β), corresponding p values, and 95% bootstrap confidence interval (*CI*).

**Supplementary Table S4. LRP1 genotype and APOE genotype interaction on MMSE scores.**

| **Factor 1** | **Factor 2** | **zMMSE** |
| --- | --- | --- |
| LRP1- | APOE ε4^-^ | 0.310±0.074 |
|  | APOE ε4^+^ | -0.669±0.245 |
| LRP+ | APOE ε4^-^ | -0.087±0.185 |
|  | APOE ε4^+^ | -0.026±0.174 |
| **ANCOVA** | ***F*** | ***p*** |
| LRP1 | 0.510 | 0.476 |
| APOE | 6.877 | 0.010 |
| LRP1*APOE | 8.546 | 0.004 |
| Age | 0.879 | 0.350 |
| Gender | 1.354 | 0.246 |
| Education | 3.268 | 0.073 |

**Note:** The z-transformed MMSE (zMMSE) values were represented as mean ± standard error of mean. Two-way analysis of covariance (ANCOVA) was applied to research the interactive effect of LRP1 and APOE on zMMSE across all subjects, with LRP1 and APOE genotypes as dichotomous fixed factors, adjusting for covariate variables of age, gender and education.

**Supplementary Figure S1 Flow chart of participant inclusion.** rsfMRI, resting-state functional MRI; CN, cognitive normal; SCD, SCD, subjective cognitive decline; MCI, mild cognitive impairment; AD, Alzheimer’s disease.

**
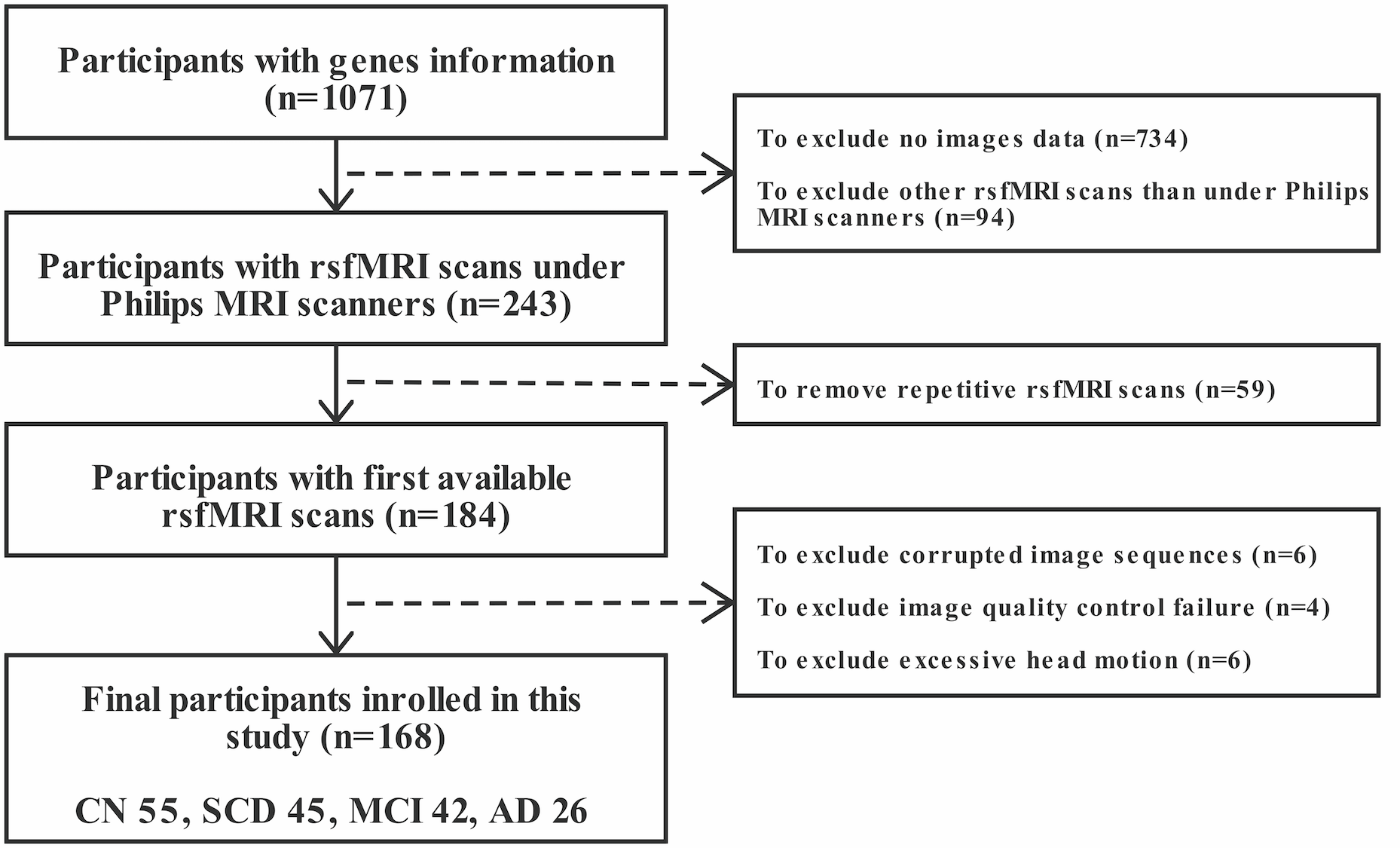
**

**Supplemental Figure S2. Spatial distribution of default mode network (DMN) identified by group independent component analysis (ICA, one-sample t-test, p < 0.001, FDR corrected).** The color bar indicates z value of functional connectivity in DMN.

**
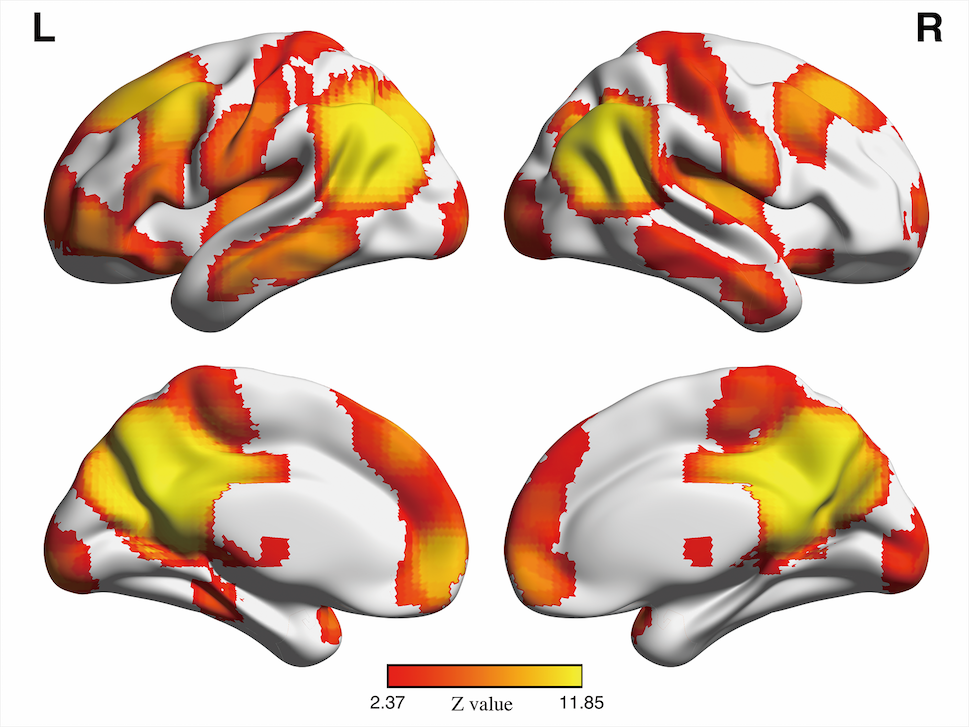
**

**References:**

1. Jessen F, Amariglio RE, van Boxtel M, et al. A conceptual framework for research on subjective cognitive decline in preclinical Alzheimer's disease. *Alzheimer's & dementia : the journal of the Alzheimer's Association*. Nov 2014;10(6):844-52.

2. McKhann G, Drachman D, Folstein M, Katzman R, Price D, Stadlan EM. Clinical diagnosis of Alzheimer's disease: report of the NINCDS-ADRDA Work Group under the auspices of Department of Health and Human Services Task Force on Alzheimer's Disease. *Neurology*. Jul 1984;34(7):939-44.

3. Faul F, Erdfelder E, Buchner A, Lang AG. Statistical power analyses using G*Power 3.1: tests for correlation and regression analyses. *Behavior research methods*. Nov 2009;41(4):1149-60.

4. Jia XZ, Wang J, Sun HY, et al. RESTplus: an improved toolkit for resting-state functional magnetic resonance imaging data processing. *Science Bulletin*. 2019;64:953–954.

5. Bell AJ, Sejnowski TJ. An information-maximization approach to blind separation and blind deconvolution. *Neural Comput*. Nov 1995;7(6):1129-59.

6. Calhoun VD, Adali T, Pearlson GD, Pekar JJ. A method for making group inferences from functional MRI data using independent component analysis. *Human brain mapping*. Nov 2001;14(3):140-51.

7. Kaneko H, Arakawa M, Funatsu K. Development of a new regression analysis method using independent component analysis. *Journal of chemical information and modeling*. Mar 2008;48(3):534-41.
